# Impact of COVID-19 on stroke admissions, treatments, and outcomes at a comprehensive stroke centre in the United Kingdom

**DOI:** 10.1007/s10072-020-04775-x

**Published:** 2020-10-06

**Authors:** Nishita Padmanabhan, Indira Natarajan, Rachel Gunston, Marko Raseta, Christine Roffe

**Affiliations:** 1 Neurosciences, Royal Stoke University Hospital, Stoke-on-Trent, UK; 2 Faculty of Medicine and Health Sciences, Keele University, Staffordshire, UK; 3 Statistics and Mathematical Modelling, Department of Molecular Genetics, Erasmus MC, Rotterdam, Netherlands

**Keywords:** Stroke, COVID-19, Thrombolysis, Thrombectomy, Infarct, Mortality

## Abstract

**Introduction:**

The coronavirus disease (COVID-19) pandemic has changed routine clinical practice worldwide with major impacts on the provision of care and treatment for stroke patients.

**Methods:**

This retrospective observational study included all patients admitted to the Royal Stoke University Hospital in Stoke-on-Trent, UK, with a stroke or transient ischaemic attack between March 15th and April 14th, 2020 (COVID). Patient demographics, characteristics of the stroke, treatment details and logistics were compared with patients admitted in the corresponding weeks in the year before (2019).

**Results:**

There was a 39.5% (*n* = 101 vs *n* = 167) reduction in admissions in the COVID cohort compared with 2019 with more severe strokes (median National Institutes of Health Stroke Scale (NIHSS) 7 vs 4, *p* = 0.02), and fewer strokes with no visible acute pathology (21.8 vs 37.1%, *p* = 0.01) on computed tomography. There was no statistically significant difference in the rates of thrombolysis (10.9 vs 13.2%, *p* = 0.72) and/or thrombectomy (5.9 vs 4.8%, *p* = 0.90) and no statistically significant difference in time from stroke onset to arrival at hospital (734 vs 576 min, *p* = 0.34), door-to-needle time for thrombolysis (54 vs 64 min, *p* = 0.43) and door-to-thrombectomy time (181 vs 445 min, p = 0.72). Thirty-day mortality was not significantly higher in the COVID year (10.9 vs 8.9%, *p* = 0.77). None of the 7 stroke patients infected with COVID-19 died.

**Conclusions:**

During the COVID-19 pandemic, the number of stroke admissions fell, and stroke severity increased. There was no statistically significant change in the delivery of thrombolysis and mechanical thrombectomy and no increase in mortality.

## Introduction

Coronavirus disease 2019 (COVID-19) is an infectious disease caused by the SARS-CoV-2 virus. As of September 21st, 2020, there are 30,675,675 confirmed COVID-19 cases and 954,417 confirmed deaths in over 216 countries/territories worldwide [1]. The United Kingdom (UK), with 390,362 confirmed cases and 41,759 confirmed deaths, continues to have community transmission at present [1]. COVID-19 is associated with a hypercoagulable state that may lead to an increase in acute cerebrovascular events with an increase in ischaemic strokes and large vessel occlusion strokes [2–4]. Preliminary global reports show a reduction in the number of stroke admissions with a delay in the presentation of acute ischaemic strokes and time to thrombolysis and thrombectomy [5–7].

The response to the coronavirus disease (COVID-19) pandemic saw organisations across the UK using varied strategies to approach changes in routine clinical practice. The Hyper Acute Stroke Unit in the Royal Stoke University Hospital also adopted a new model. Outpatient clinics with direct patient contact were closed and replaced by virtual clinics. All holidays, study leave and non-clinical activity were cancelled, thereby increasing the shop floor presence of nursing and medical workforce. The junior doctor schedule was changed from the standard 9 am to 5 pm working hours to a more widely distributed 24 h round the clock availability on the ward with reduced additional speciality responsibilities.

The UK went into lockdown on March 23rd, 2020, and the COVID-19 pandemic began in Stoke-on-Trent in the West Midlands around March 15th, 2020. Our study aimed to establish whether and how the delivery of stroke care during this period was affected by the COVID-19 pandemic. We did this by comparing 4 weeks when we had the highest number of COVID-19 cases in 2020 with the same period in the year before. The aim of this was to see whether there were any changes to the number of stroke unit admissions in the COVID year.

## Methods

### Study design and setting

This retrospective observational study was based at the Hyper Acute Stroke Unit (HASU) in the Royal Stoke University Hospital, Stoke-on-Trent, UK. The 32-bedded comprehensive tertiary stroke centre at the Royal Stoke University Hospital in Stoke-on-Trent caters to a local population of half a million individuals. We receive secondary referrals from three district general hospitals and tertiary referrals for mechanical thrombectomy from four acute stroke units. Situated in a region with a high prevalence of vascular co-morbidity due to high unemployment, the unit has a secondary catchment area of 1.5 million and a tertiary catchment area of 3 million with around 1250 stroke admissions every year.

Two cohorts were identified for this study from local Sentinel Stroke National Audit Programme data. The Sentinel Stroke National Audit Programme database records all patients admitted to the hospital with a stroke diagnosis. This includes patients who are not admitted to the Hyper Acute Stroke Unit. Cohort 1 (COVID) included all patients admitted to the hospital during the first 4 weeks of the COVID-19 pandemic with a diagnosis of stroke/stroke mimic/transient ischaemic attack (TIA), i.e. between March 15th and April 14th, 2020. All patients admitted with a stroke received care on the Hyper Acute Stroke Unit during this period. Cohort 2 (pre-COVID) included all patients admitted to the hospital during the corresponding weeks in the previous year, i.e. between March 15th and April 14th, 2019. Patients who were not admitted with a diagnosis of stroke/TIA/stroke mimic were excluded.

### Data collection

Data on patient demographics, characteristics of stroke, the time between onset and hospital presentation, treatment details and outcomes were included in this study analysis. Data were extracted from the local Sentinel Stroke National Audit Programme database and from the hospital’s electronic health records system. This includes discharge letters, blood results and imaging results. The ward admissions’ book and patient notes were also accessed to gather information.

### Plan of analysis/statistical tools

Data was entered and summarised in Microsoft Excel. Categorical variables were compared by means of *Z* test (binary variables), Chi-squared test (categorical variables with 3 or more categories) and Fisher’s exact test (low expected per-cell observations). The Kolmogorov-Smirnov test was used to assess normality of continuous variables (age, National Institutes for Health Stroke Scale (NIHSS) score and timings) which were subsequently compared using Student *t* test or Mann-Whitney *U* test for normally distributed and non-normal data, respectively. Statistical analysis was done in R statistical software tool (R Core Team, 2018). Significance was accepted at *p* < 0.05.

## Results

There were 101 and 167 admissions with stroke/TIA/stroke mimic in the COVID cohort and the pre-COVID cohort, respectively, representing 39.5% fewer admissions during the COVID pandemic peak (Table 1). In the COVID cohort, 7 (6.9%) were positive for SARS-CoV-2 virus on polymerase chain reaction testing. No tests were done in 2019 (Table 1). The mean age of patients was 70.4 SD 14.9/73.2 SD 13.8 years in the COVID and pre-COVID cohorts, with just under 50% females (47.5 vs 49.1%) and a high proportion of White ethnicity (90.1 vs 88.6%). There was no statistically significant difference in any of these characteristics between the two cohorts (Table 2).

**Table 1:** Comparison of stroke admissions during COVID year and pre-COVID year

	COVID	Pre-COVID
Number of stroke admissions	101	167
COVID-19 positive (*n* (%))	7 (6.9%)	N/A
COVID-19 mortality (*n* (%))	0 (0.0%)	N/A

*n*, number of patients

**Table 2:** Demographic details of stroke admissions in the COVID and pre-COVID cohorts

Demographics	COVID (*n* = 101)	Pre-COVID (*n* = 167)	*p* value
Age mean (SD)	70.4 (14.9)	73.2 (13.8)	0.13^a^
Female sex (*n* (%))	48 (47.5%)	82 (49.1%)	0.90^b^
Ethnicity White (*n* (%))	91 (90.1%)	148 (88.6%)	0.86^b^
Ethnicity Non-White/not specified (*n* (%))	10 (9.9%)	19 (11.4%)	

*n*, number of patients; *SD*, standard deviation

^a^*t* test

^b^*Z* test

The final diagnosis was stroke (ischaemic or haemorrhagic) in 92 (91.1%)/145 (86.8%), transient ischemic attack in 0 (0%)/2 (1.2%) and stroke mimic in 9 (8.9%)/20 (12.0%) in the COVID and pre-COVID cohorts, respectively. Among the strokes, 70 (69.4%)/121 (72.2%) were ischaemic, and 22 (21.8%)/24 (14.4%) were haemorrhagic in the two respective cohorts. None of the differences was statistically significant (Table 3).

**Table 3:** Characteristics of stroke in the COVID and pre-COVID cohorts

*n* (%) except where indicated	COVID (*n* = 101)	Pre-COVID (*n* = 167)	*p* value
Final diagnosis			
Stroke (ischemic or haemorrhagic)	92 (91.1%)	145 (86.8%)	0.39^a^
Transient ischaemic attack	0 (0%)	2 (1.2%)	0.53^d^
Stroke mimic	9 (8.9%)	20 (12.0%)	0.56^a^
Radiological findings			
Visible acute infarct on imaging	57 (56.4%)	81 (48.5%)	0.26^a^
Primary intracerebral haemorrhage	22 (21.8%)	24 (14.4%)	0.16^a^
No acute ischemia or haemorrhage	22 (21.8%)	62 (37.1%)	0.01^a^
Stroke severity			
NIHSS (median (IQR))	7 (3–16)	4 (2–12)	0.02^b^
Mild stroke (NIHSS 0–3)	31 (30.7%)	71 (42.5%)	0.14^c^
Moderate stroke (NIHSS 4–8)	27 (26.7%)	43 (25.8%)	
Severe stroke (NIHSS > 8)	43 (42.6%)	53 (31.7%)	
Bamford classification			
Total anterior circulation infarct	21 (20.8%)	32 (19.1%)	0.87^a^
Partial anterior circulation infract	15 (14.9%)	35 (20.9%)	0.28^a^
Lacunar infarct	21 (20.8%)	44 (26.3%)	0.38^a^
Posterior circulation infarct	13 (12.9%)	10 (5.9%)	0.08^a^
Haemorrhagic stroke	22 (21.8%)	24 (14.4%)	0.16^a^

*IQR*, interquartile range; *n*, number of patients; *NIHSS*, National Institutes of Health Stroke Scale

^a^*Z* test

^b^Mann-Whitney *U* test

^c^Chi-squared test

^d^Fisher’s exact test

Stroke severity was significantly higher in the COVID cohort than in the pre-COVID cohort (median (IQR) NIHSS 7 (3–16) vs 4 (2–12), *p* = 0.02). Significantly fewer patients had no acute findings (ischaemia or haemorrhage) on imaging in the COVID cohort than pre-COVID (22 (21.8%) vs 62 (37.1%), *p* = 0.01) (Table 3).

There were no statistically significant differences in the rates of thrombolysis (11 (10.9%) vs 22 (13.2%)) and mechanical thrombectomy (6 (5.9%) vs 8 (4.8%)) between the COVID and pre-COVID cohorts. Moreover, no statistically significant difference was found between cohorts in the time from onset to hospital arrival (median 734 vs 576 min), door to thrombolysis time (mean 54 vs 64 min) and door to groin time for mechanical thrombectomy (mean 181 vs 445 min) (Table 4).

**Table 4:** Impact of COVID-19 on frequency and timing of thrombolysis and mechanical thrombectomy

	COVID (*n* = 101)	Pre-COVID (*n* = 167)	*p* value
Thrombolysis (*n* (%))	11 (10.9%)	22 (13.2%)	0.72^a^
Mechanical thrombectomy (*n* (%))	6 (5.9%)	8 (4.8%)	0.90^a^
Time from onset to arrival (median (IQR)) min	734 (246–1091)	576 (128–1197)	0.34^b^
Time from arrival to thrombolysis (mean SD) min	54 (30)	64 (47)	0.43^c^
Time from arrival to thrombectomy (mean SD) min	181 (62)	445 (566)	0.72^c^

IQR = interquartile range; n = number of patients; SD = standard deviation

^a^*Z* test

^b^Mann*-*Whitney *U* test

^c^*t* test

Thirty-day mortality was similar between the COVID and pre-COVID cohorts (11 (10.9%) vs 15 (8.9%), *p* = 0.77). There were no deaths among COVID-positive patients, and there was no statistically significant difference in stroke recurrence (4 (3.9%) vs 13 (7.8%), *p* = 0.32) and discharge destination between the cohorts (Table 5).

**Table 5:** Impact of COVID-19 on stroke outcome

Outcome *n* (%)	COVID (*n* = 101)	Pre-COVID *n* = 167	*p* value
30-day mortality	11 (10.9%)	15 (8.9%)	0.77^a^
Stroke recurrence	4 (3.9%)	13 (7.8%)	0.32^a^
Discharged to home/care home	59 (58.4%)	106 (63.5%)	0.28^b^
Discharged to another acute setting	17 (16.8%)	36 (21.5%)	
Discharged to an inpatient rehabilitation centre	15 (14.9%)	14 (8.4%)	
Death during hospital stay	10 (9.9%)	11 (6.6%)	

*n*, number of patients

^a^*Z* test

^b^2x4 Chi-squared test

## Discussion

The key findings of this project are a reduction in the total number of stroke admissions and an increase in stroke severity during the COVID-19 pandemic. There was no statistically significant change in the rates and timings of delivery of thrombolysis and mechanical thrombectomy, and no worsening of stroke outcomes during the pandemic.

During the same time periods, the West Midlands Ambulance Service University NHS Foundation Trust Ambulance Service, which covers the Royal Stoke University Hospital and the wider West Midlands region, reported no reduction in callouts for stroke [8]. While this data suggests that the same number of people are calling 999 with acute stroke symptoms, fewer are admitted with a diagnosis of stroke. It is possible that patients with milder symptoms were treated on-site rather than taken to the hospital. We have no details to confirm this. However, our observation of greater stroke severity in admitted patients lends some support to this possibility. Patients who did not present to the hospital were not captured as there is no community stroke registry.

Fewer callouts resulting in a transfer to a hospital may have been due to national guidance to avoid hospital admission, wherever possible, or because of patients refusing transfer to hospital because of fear of COVID-19. Rudilosso et al. report an increase in daily emergency phone calls with a fall in the number of stroke admissions during their COVID peak in Barcelona [9]. In contrast, teams from Seville and Ohio report significant decreases in their daily stroke alerts and telephone stroke consultations [5, 10]. The reason for these differences between centres is not apparent. Zhao et al. report a significant drop in the hospital admissions for stroke during COVID-19 pandemic in China [11]. A multicentre study by Hoyer et al. in Germany reports a significant reduction in acute stroke and TIA admissions following the implementation of national lockdown and decreased public mobility [12].

In our cohort, stroke patients presented with a higher median NIHSS with no statistically significant difference in the stroke demographics, i.e. age, sex and ethnicity. This is different from Rudilosso et al., who report younger strokes, but no difference in severity at presentation [9].

We did not find a significant increase in the time between symptom onset and arrival at the hospital. This mirrors findings from Ohio and Barcelona [5, 9]. However, Schirmer et al. and Montaner et al. describe a significant increase in stroke onset time to arrival at the hospital during the COVID-19 period for a collaboration of 12 hospitals in 6 states in the USA and Seville in Spain, respectively [6, 10]. This is similar to Frisullo et al., who reported a significant increase in the onset-to-door time in the early phase of the pandemic in Rome [13]. Such differences may reflect different policies in prioritisation of stroke transfer and/or differing strain on existing services due to high numbers of COVID-19 patients.

In our study, neither the proportion of patients treated with thrombolysis and/or thrombectomy nor the timeliness of interventions differed significantly to the year before the COVID pandemic. However, numbers were small, making point estimates imprecise. Lower rates of intravenous thrombolysis and thrombectomy were reported from Spain and China where their health services were overrun [9–11]. A study conducted in France showed significant delays between imaging and groin puncture timings during the COVID-19 period. However, this difference was not significant when patients underwent thrombectomy at the first hospital that they presented to [7]. A study of 17 thrombectomy centres in Europe, Canada and the USA showed a significant decrease in the number of thrombectomies performed and an increase in stroke onset-to groin puncture after confinement measures were put in place [14].

In the UK, the medical service was not overwhelmed, but we did have higher mortality in the COVID year. A pooled analysis by Aggarwal shows increased odds of severe cerebrovascular disease in COVID-19-infected patients [15]. This is unlike our experience. Stroke mortality has not increased despite greater severity, and there was no statistically significant change in outcomes at discharge. We have been able to maintain quality care with comparable outcomes and without significant mortality.

It can be hypothesised that a lower number of stroke admissions allowed us to provide a better service to the individual. In addition, cancellation of all non-clinical tasks, annual and study leave compensated for staff absences through COVID-19 infection and quarantine, freed up consultant time and increased consultant presence on the ward. Junior doctor workforce was more evenly distributed throughout the 24 hours, and this may have allowed for earlier detection and treatment of complications. The greater presence of medical staff translated to lower threshold for nurses to contact doctors of all grades for advice which led to focussed assessments and improved patient care. While service was provided as usual for patients who presented to the hospital, secondary prevention may have been missed out for patients who did not present themselves. For future pandemics, this needs to be managed proactively by raising public awareness, liaison with ambulance services, and general practitioners, and the provision of ‘catch-up’ services to maintain secondary prevention and prevent avoidable strokes.

Locally, the change in number and severity of stroke patients prompted a public awareness campaign via local Stoke-on-Trent news and radio interviews during the month of May highlighting concerns over the drop in stroke admissions in our region and advising the public to seek help from the National Health Service (NHS) if they experience symptoms of a stroke [16]. There was a special focus on people with minor stroke symptoms and elderly isolated patients. A week later, the National Health Service ‘Help Us Help You’ campaign by the British Government also urged the public to continue to act FAST (face, arms, speech, time) by calling 999 for stroke symptoms [17].

The main limitation of our study is that it is from a single hospital. Numbers are therefore too small to detect small, but potentially important differences reliably. It will be important to see whether our findings are reflected elsewhere in the UK and whether the trend of admissions is due to fewer patients presenting, or fewer patients who call 999 being admitted to hospital.

## Conclusion

We provide evidence that stroke admissions have reduced by two-fifths during the COVID-19 pandemic, but that admitted stroke patients had greater neurological deficits. Rate of delivery of hyperacute interventions such as thrombolysis and mechanical thrombectomy was unchanged, and there was no increase in mortality or adverse discharge outcomes. Our findings contribute to the knowledge base of the effect of service changes on outcomes in times of exceptional stress on the health system.

## Data Availability

The datasets analysed during the current study are available from the corresponding author on reasonable request.
